# Novel Projections to the Cerebrospinal Fluid-Contacting Nucleus From the Subcortex and Limbic System in Rat

**DOI:** 10.3389/fnana.2020.00057

**Published:** 2020-08-20

**Authors:** Si-Yuan Song, Xiao-Meng Zhai, Jia-Hao Dai, Lei-Lei Lu, Cheng-Jing Shan, Jia Hong, Jun-Li Cao, Li-Cai Zhang

**Affiliations:** Jiangsu Province Key Laboratory of Anesthesiology, Xuzhou Medical University, Xuzhou, China

**Keywords:** CSF-contacting nucleus, subcortex, limbic system, projection, retrograde trace

## Abstract

**Objective**: To identify the novel projections received by the cerebrospinal fluid (CSF)-contacting nucleus from the subcortex and limbic system to understand the biological functions of the nucleus.

**Methods**: The cholera toxin subunit B (CB), a retrograde tracer, was injected into the CSF-contacting nucleus in Sprague–Dawley rats. After 7–10 days, the surviving rats were perfused, and the whole brain and spinal cord were sliced for CB immunofluorescence detection. The CB-positive neurons in the subcortex and limbic system were observed under a fluorescence microscope, followed by 3D reconstructed with the imaris software.

**Results**: CB-positive neurons were found in the basal forebrain, septum, periventricular organs, preoptic area, and amygdaloid structures. Five functional areas including 46 sub-regions sent projections to the CSF-contacting nucleus. However, the projections had different densities, ranging from sparse to moderate, to dense.

**Conclusions**: According to the projections from the subcortex and limbic system, we hypothesize that the CSF-contacting nucleus participates in emotion, cognition, homeostasis regulation, visceral activity, pain, and addiction. In this study, we illustrate the novel projections from the subcortex and limbic system to the CSF-contacting nucleus, which underlies the diverse and complicated circuits of the nucleus in body regulations.

## Introduction

The cerebrospinal fluid (CSF)-contacting nucleus is a special nucleus recently identified in the brain. It is located within the pons including the isthmic region (Song et al., 2019). The unique characteristic of this nucleus is that its axons form bundles and go across the CSF-brain barrier stretching into the CSF (Song and Zhang, 2018; Song et al., 2019). After injecting the tracer CB-HRP or cholera toxin subunit B (CB) into the ventricle, only the CSF-contacting nucleus in the brain parenchyma is labeled (Song et al., 2019). Our previous studies have demonstrated the connections of the CSF-contacting nucleus with non-CSF-contacting neurons, glial cells, and blood vessels (Zhang et al., 2003). It implies that the CSF-contacting nucleus may be considered as an important structure bridging the brain and CSF, or that it plays an extremely important role in physiological activities. Moreover, our recent studies reveal that this nucleus receives the projections from the hypothalamus (Song et al., 2020b) and brainstem (Song et al., 2020a), which implies that the CSF-contacting nucleus participates in complex and diverse neural circuits modulating different behaviors. The relationships with some of the life activities such as pain (Liu et al., 2017), stress (Wu et al., 2015), and drug addiction (Lu et al., 2011) have been already reported.

The present study aimed to identify if these CSF-contacting nucleus functions are underpinned by monosynaptic projections from the subcortex and limbic system. The subcortex and limbic system are located below the cerebrum rostral to the diencephalon. These two brain regions, which include the basal forebrain, septum, preoptic area, amygdaloid structures, which play important roles in modulating the body activities. For example, the basal forebrain, septum, and amygdaloid structures are known to modulate cognition (Talishinsky and Rosen, 2012; Aitta-Aho et al., 2018; Chaves-Coira et al., 2018), emotion (Sah et al., 2003; Talishinsky and Rosen, 2012; Zhang et al., 2018), and autonomic nervous functions (Loewy, 1991; Sah et al., 2003; Talishinsky and Rosen, 2012). The preoptic area plays essential roles in fluid balance (Augustine et al., 2018), body temperature regulation (Abbott and Saper, 2018; Mohammed et al., 2018), and autonomic functions (Fassini et al., 2017).

In this study, we mapped the novel projections from the subcortex and limbic system to the CSF-contacting nucleus by using the retrograde tracing method. The biological functions of the CSF-contacting nucleus can be speculated according to the projections, which will lay the foundations for further deeper research.

## Materials and Methods

### Experimental Animals

Fifteen specific pathogen-free male Sprague–Dawley rats (weight 250 ± 50 g) were acquired from the Experimental Animal Center of Xuzhou Medical University. Rats successfully injected with the tracer into the CSF-contacting nucleus were used for observation and analysis (*n* = 6). All animal experiments were approved by and performed following the guidelines of the Committee for Ethical Use of Laboratory Animals of Xuzhou Medical University.

### Retrograde Tracer Injection

The rats were first anesthetized with pentobarbital sodium (40 mg/kg, i.p.). Then, the head of the animal was fixed on the stereotaxic instrument (Stoelting 51700, Wood Dale, IL, USA) and 0.2 μl of the retrograde tracer (1% CB) solution (Cat# abs80001, Absin, China) was injected according to the CSF-contacting nucleus stereotaxic coordinates (Bregma: 8.24 ± 0.18 mm, Lateral: 0.09 ± 0.01 mm, Depth: 6.45 ± 0.11 mm; Song et al., 2019). A Hamilton syringe (Hamilton Company, Switzerland) with a 33G needle tip controlled by a microinfusion pump (KD Scientific, Holliston, MA, USA) was used for the CB solution injections. The injections were made for about 30 min, and the syringe was left in place for 10–15 min before retraction.

### Sampling and Sectioning

After 7–10 days, the surviving rats were perfused. The rats were first anesthetized with pentobarbital sodium (40 mg/kg, i.p.) and then perfused with 300 ml of 0.01 M phosphate-buffered saline (pH 7.4), followed without interruption by 300 ml of 4% paraformaldehyde in 0.2 M phosphate buffer (pH 7.4). The whole brain and spinal cord were isolated and sectioned coronally into 40-μm slices on a cryostat (CM1900, Leica, Germany). In this study, only the subcortex and limbic system were captured and analyzed.

### Tracer Immunofluorescence Staining and Image Acquisition

All the sections were subjected to CB immunofluorescence staining. The antisera used for immunofluorescence processing were diluted in a solution of 0.1 M PBS containing 0.3% Triton X-100. The sections were incubated in rabbit anti-CB primary antibody (1:600, Cat# ab34992, Abcam) for 48 h at 4°C. Next, the slices were incubated in donkey anti-rabbit Alexa Fluor 488 secondary antibody (1:200, Cat# A-21206, Thermo Fisher) for 2 h in the dark at room temperature. Then, the sections were mounted in sequence onto slides, counterstained with 4’,6-diamidino-2-phenylindole (DAPI), and covered with coverslips. Images of the sections of the subcortex and limbic system were captured under a fluorescence microscope (DM6, Leica, Germany) and confocal laser microscope (Zeiss, Germany). The 10× lens was used to capture the positive neurons, while the 40× lens was applied to show the detailed structures.

### Three-Dimensional Reconstruction of the Subcortex and Limbic System Projections

The CB-positive neurons were aligned, segmented, and registered on a common rat reference atlas (Paxinos and Watson, 2007). The three-dimensional (3D) subcortex and limbic system projections were reconstructed using Imaris software version 8.4.1 (Bitplane, USA).

### Statistics

SPSS 13.0 software was used for data analysis in the present study. Data were presented as mean ± SD. The cell density of CB-positive neurons (cell number/0.2 mm^2^ area) in each brain region was calculated using Image-Pro Plus 7.0 software, and then classified according to the following densities: <5, sparse; 6–10, moderate; and >10, dense.

## Results

### Injection of the Tracer Into the CSF-Contacting Nucleus

**Figure 1:**
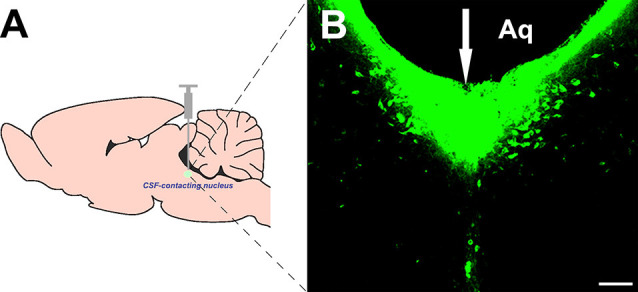
Injection site of cholera toxin subunit B (CB) into the cerebrospinal fluid (CSF)-contacting nucleus **(A,B)**. White arrow (↓): site of the CB injection. Aq, aqueduct. Bar = 100 μm.

The retrograde tracer CB was injected directly into the CSF-contacting nucleus according to the stereotaxic coordinates (Figure 1A). The tracer produced dense green immunofluorescence-positive staining within the CSF-contacting nucleus (Figure 1B).

### Cellular Morphology of the Subcortex and Limbic System Projections

**Figure 2:**
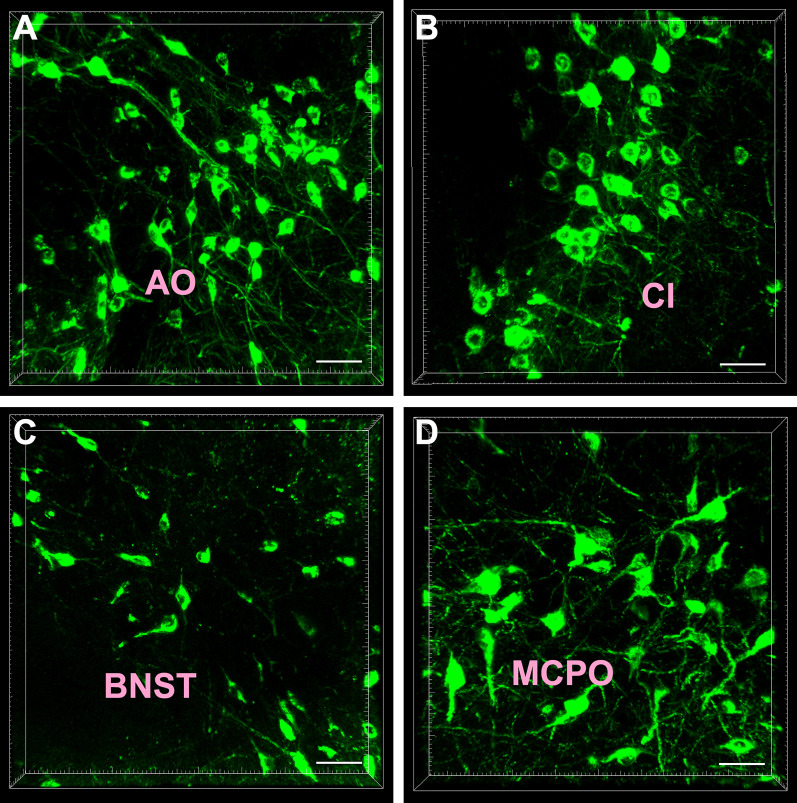
Cellular morphology of the cholera toxin subunit B-positive neurons. The representative photographs were captured from the anterior olfactory nucleus (AO; **A**) claustrum (Cl; **B**) bed nucleus of the stria terminalis (BNST; **C**) and magnocellular preoptic nucleus (MCPO; **D**). Bar = 40 μm.

Most of the CB-positive neurons in the subcortex and limbic system appeared round or fusiform in shape. The neurons were of different sizes, and the processes were sparse and short. In the anterior olfactory nucleus (AO) and magnocellular preoptic nucleus (MCPO) of the preoptic area, the CB-positive neurons were mainly large in size and had many processes. Among them, 1–2 processes were longer and reached towards other parts of the brain (Figure 2).

### Projection Sites of the Subcortex and Limbic System

The retrogradely labeled neurons were located in five functional areas including 46 sub-regions in the subcortex and limbic system. However, their density ranged from being sparse or moderate to strong in each functional region.

**Figure 3:**
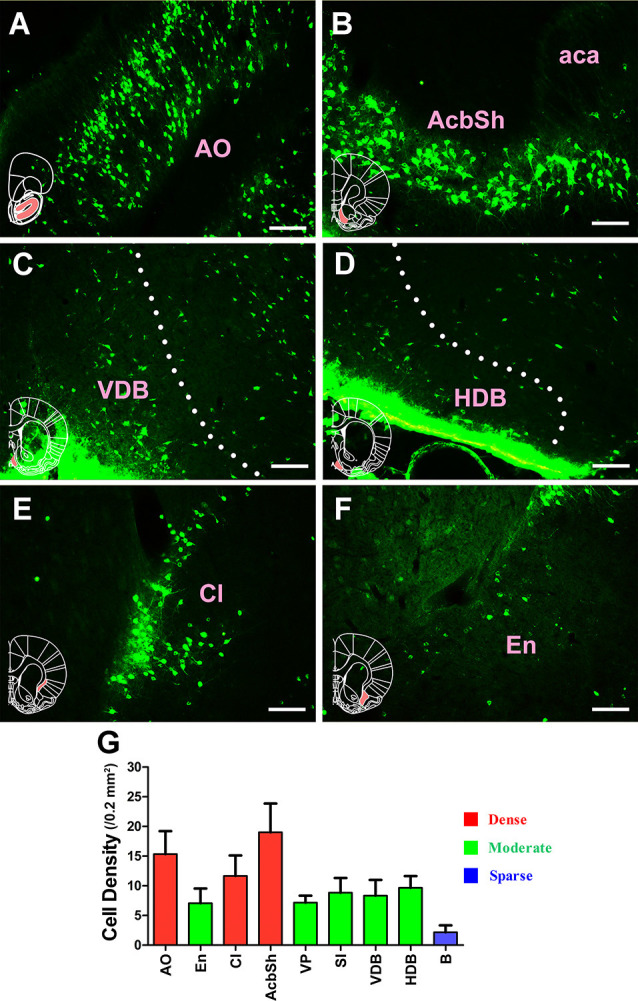
Distribution of cholera toxin subunit B-positive neurons in the basal forebrain. **(A–F)** The positive neurons in AO, AcbSh, VDB, HDB, Cl, and En. **(G)** Statistics of the input amounts of cholera toxin subunit B-positive cells from the basal forebrain to the CSF-contacting nucleus (mean ± SD, *n* = 6; AO, anterior olfactory nucleus; aca, anterior commissure anterior part; AchSh, accumbens nucleus shell; VDB, nucleus of the vertical limb of the diagonal band; HDB, nucleus of the horizontal limb of the diagonal band; Cl, claustrum; En, endopiriform nucleus). Bar = 100 μm.

The projections from the entire subcortex and limbic system to the CSF-contacting nucleus could be identified by the positively labeled neurons. In the basal forebrain, nine sub-regions had projections of the CSF-contacting nucleus. The AO, claustrum, and accumbens nucleus shell (AcbSh) had strong projections to the CSF-contacting nucleus (cell density: AO 15.33 ± 3.87, Cl 11.67 ± 3.44, and AcbSh 19 ± 4.86). The endopiriform nucleus (En), ventral pallidum (VP), substantia innominata (SI), the nucleus of the vertical limb of the diagonal band (VDB), and nucleus of the horizontal limb of the diagonal band (HDB) had moderate projections (cell density: En 7.06 ± 2.48, VP 7.17 ± 1.17, SI 8.83 ± 2.48, VDB 8.33 ± 2.66, and HDB 9.67 ± 1.97), whereas the basal nucleus (B) had sparse projections to the CSF-contacting nucleus (cell density: B 2.17 ± 1.17; Figure 3).

**Figure 4:**
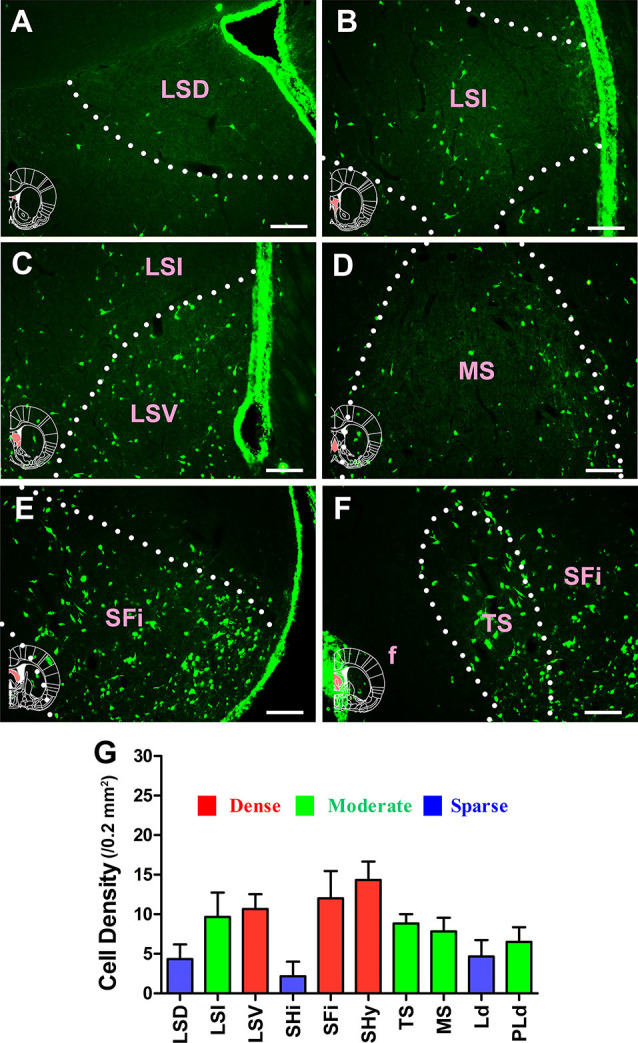
Distribution of cholera toxin subunit B-positive neurons in the septum. **(A–F)** The positive neurons in LSD, LSI, LSV, MS, SFi, and TS. **(G)** Statistics of the input amounts of cholera toxin subunit B-positive cells from the septum to the CSF-contacting nucleus (mean ± SD, *n* = 6; LSD, lateral septal nucleus dorsal part; LSI, lateral septal nucleus intermediate part; LSV, lateral septal nucleus ventral part; MS, medial septal nucleus; SFi, septofimbrial nucleus; TS, triangular septal nucleus; f, fornix). Bar = 100 μm.

In the septum, 10 sub-regions had projections of the CSF-contacting nucleus. CB-positive neurons were found in the lateral septal nucleus dorsal part (LSD); lateral septal nucleus intermediate part (LSI); lateral septal nucleus ventral part (LSV); septohippocampal nucleus (SHi); septofimbrial nucleus (SFi); septohypothalamic nucleus (SHy); triangular septal nucleus (TS); medial septal nucleus (MS); lambdoid septal zone (Ld); and paralambdoid septal nucleus (PLd). Among these, the LSV, SFi, and SHy sent strong and dense projections (cell density: LSV 10.67 ± 1.86, SFi 12 ± 3.46, and SHy 14.33 ± 2.34); the LSI, TS, MS, and PLd sent moderate projections (cell density: LSI 9.67 ± 3.08, TS 8.33 ± 1.17, MS 7.83 ± 1.72, and PLd 6.5 ± 1.87); and the LSD, SHi, and Ld sent sparse projections to the CSF-contacting nucleus (cell density: LSD 4.33 ± 1.86, SHi 2.17 ± 1.83, and Ld 4.67 ± 2.07; Figure 4).

**Figure 5:**
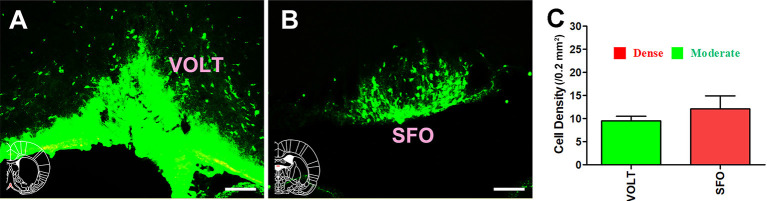
Distribution of cholera toxin subunit B-positive neurons in the circumventricular organs. **(A,B)** The positive neurons in VOLT and SFO. **(C)** Statistics of the input amounts of cholera toxin subunit B-positive cells from the circumventricular organs to the CSF-contacting nucleus (mean ± SD, *n* = 6; VOLT, vascular organ of the lamina terminalis; SFO, subfornical organ). Bar = 100 μm.

For the circumventricular organs, the vascular organ of the lamina terminalis (VOLT) sent moderate projections to the CSF-contacting nucleus, whereas the subfornical organ (SFO) sent strong projections (cell density: VOLT 9.5 ± 1.05 and SFO 12.17 ± 2.71; Figure 5).

**Figure 6:**
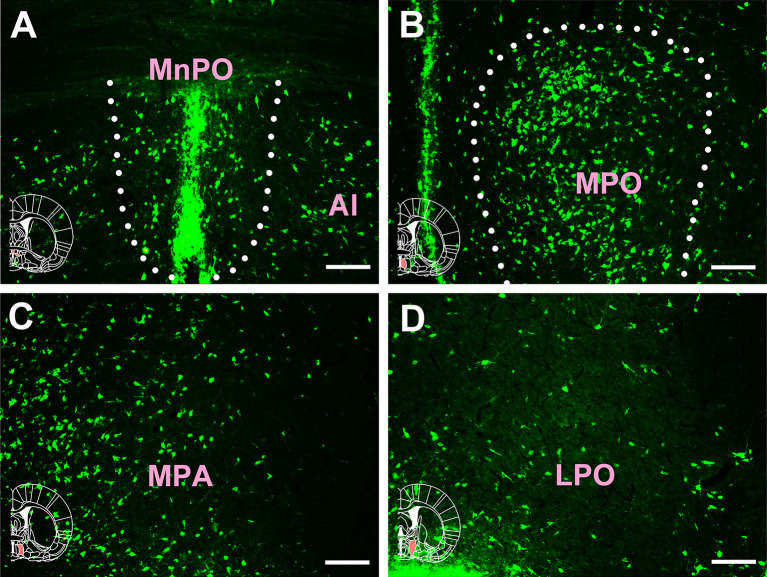
Distribution of cholera toxin subunit B-positive neurons in the preoptic area Part I. **(A–D)** The positive neurons in MnPO, Al, MPO, MPA, and LPO (MnPO, median preoptic nucleus; Al, alar nucleus; MPO, medial preoptic nucleus; MPA, medial preoptic area; LPO, lateral preoptic area). Bar = 100 μm.

**Figure 7:**
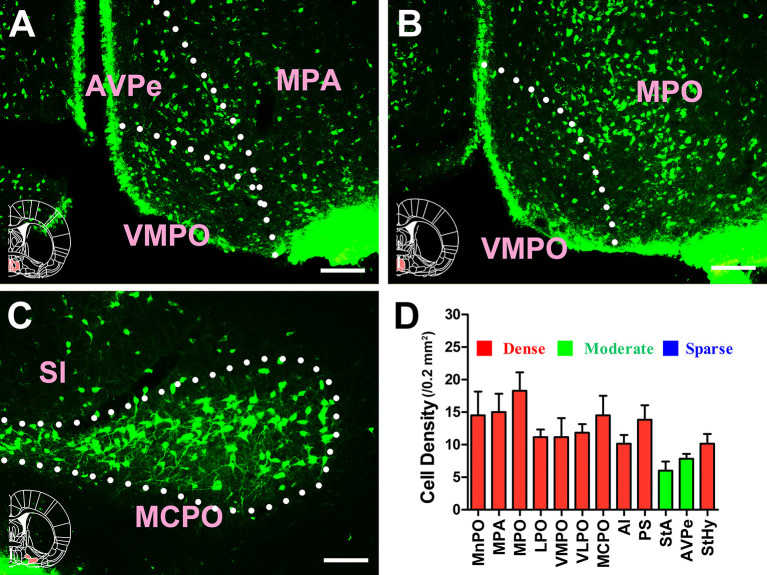
Distribution of cholera toxin subunit B-positive neurons in the preoptic area Part II. **(A–C)** The positive neurons in AVPe, VMPO, MPA, MPO, MCPO, and SI. **(D)** Statistics of the input amounts of cholera toxin subunit B-positive cells from the preoptic area to the CSF-contacting nucleus (mean ± SD, *n* = 6; MPA, medial preoptic area; MPO, medial preoptic nucleus; AVPe, anteroventral periventricular nucleus; VMPO, ventromedial preoptic nucleus; SI, substantia innominata; MCPO, magnocellular preoptic nucleus). Bar = 100 μm.

Extensive and plentiful CB-positive neurons could be found in the preoptic area, where 12 sub-regions had projections to the CSF-contacting nucleus. CB-positive neurons were found in the median preoptic nucleus (MnPO), medial preoptic area (MPA), medial preoptic nucleus (MPO), lateral preoptic area (LPO), ventromedial preoptic nucleus (VMPO), ventrolateral preoptic nucleus (VLPO), MCPO, alar nucleus (Al), parastrial nucleus (PS), strial part of the preoptic area (StA), anteroventral periventricular nucleus (AVPe), and striohypothalamic nucleus (StHy). Among these, the MnPO, MPA, MPO, LPO, VMPO, VLPO, MCPO, Al, PS, and StHy sent strong projections to the CSF-contacting nucleus (cell density: MnPO 14.5 ± 3.67, MPA 15 ± 2.83, MPO 18.28 ± 2.85, LPO 11.17 ± 1.17, VMPO 11.17 ± 2.93, VLPO 11.83 ± 1.33, MCPO 14.5 ± 3.02, Al 10.17 ± 1.33, PS 13.83 ± 2.23, and StHy 10.17 ± 1.47), whereas the StA and AVPe sent moderate projections (cell density: StA 6 ± 1.41 and AVPe 7.83 ± 0.75; Figures 6, 7).

**Figure 8:**
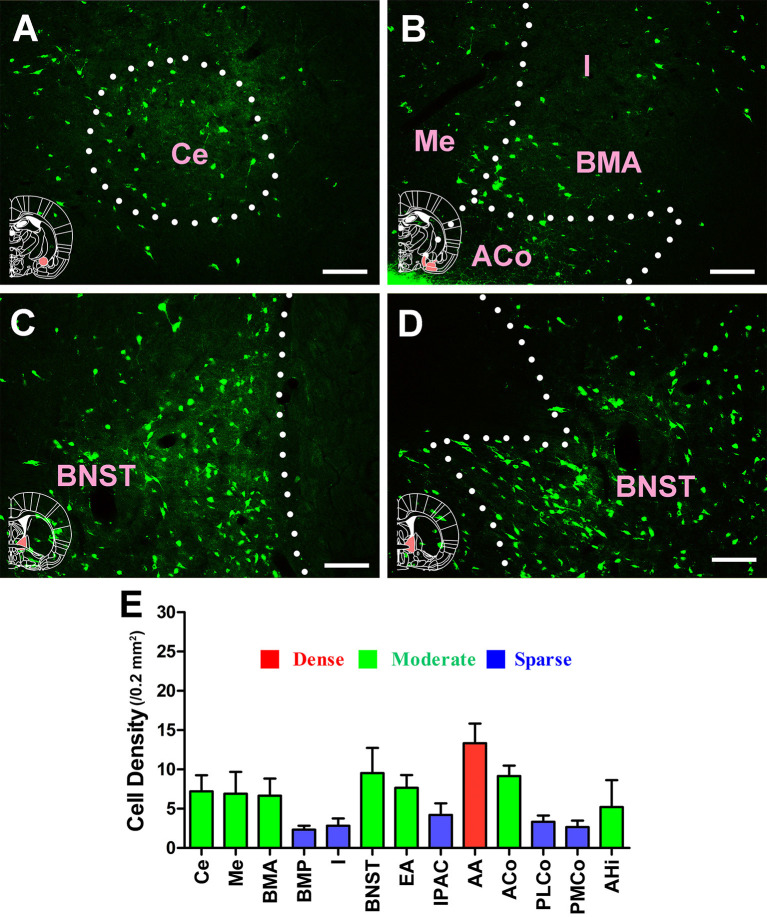
Distribution of cholera toxin subunit B-positive neurons in the amygdaloid structures. **(A–D)** The positive neurons in Ce, Me, I, BMA, ACo, and BNST. **(E)** Statistics of the input amounts of cholera toxin subunit B-positive cells from the amygdaloid structures to the CSF-contacting nucleus (mean ± SD, *n* = 6; Ce, central amygdaloid nucleus; Me, medial amygdaloid nucleus; I, intercalated nuclei of the amygdala; BMA, basomedial amygdaloid nucleus anterior part; ACo, anterior cortical amygdaloid nucleus; BNST, bed nucleus of the stria terminalis). Bar = 100 μm.

In the amygdaloid structures, 13 sub-regions had projections of the CSF-contacting nucleus. CB-positive neurons were detected in the central amygdaloid nucleus (Ce), medial amygdaloid nucleus (Me), basomedial amygdaloid nucleus anterior part (BMA), basomedial amygdaloid nucleus posterior part (BMP), intercalated nuclei of the amygdala (I), bed nucleus of the stria terminalis (BNST), sublenticular extended amygdala (EA), interstitial nucleus of the posterior limb of the anterior commissure (IPAC), anterior amygdaloid area (AA), anterior cortical amygdaloid nucleus (ACo), posterolateral cortical amygdaloid nucleus (PLCo), posteromedial cortical amygdaloid nucleus (PMCo), and amygdalohippocampal area (AHi). The AA sent strong projections to the CSF-contacting nucleus (cell density: AA 13.33 ± 2.5), whereas the Ce, Me, BMA, EA, ACo, and AHi sent moderate projections (cell density: Ce 7.22 ± 2.05, Me 6.92 ± 2.76, BMA 6.67 ± 2.16, EA 7.67 ± 1.61, ACo 9.17 ± 1.33, and AHi 5.22 ± 3.42), and the BMP, I, IPAC, PLCo, and PMCo sent sparse projections (cell density: BMP 2.33 ± 0.52, I 2.83 ± 0.94, IPAC 4.22 ± 1.48, PLCo 3.33 ± 0.82, and PMCo 2.67 ± 0.82; Figure 8).

### 3D Reconstruction of the Subcortex and Limbic System Projections

**Figure 9:**
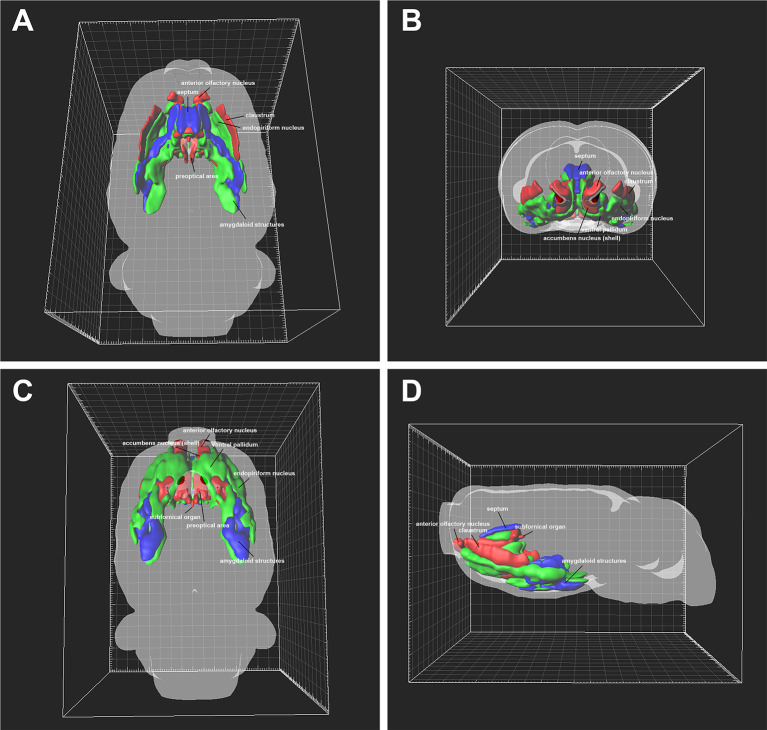
Three-dimensional view of the projection patterns from the subcortex and limbic system to the cerebrospinal fluid (CSF)-contacting nucleus. **(A)** Dorsal view, **(B)** anterior view, **(C)** ventral view, and **(D)** lateral view. The red areas represent strong projections, the green areas represent moderate projections, and the blue areas represent weak projections to the CSF-contacting nucleus.

The densities of the subcortex and limbic system projections were obvious in the 3D view, where the red areas represent highly dense projections (AO, Cl, AcbSh, LSV, SFi, SHy, SFO, MnPO, MPA, MPO, LPO, VMPO, VLPO, MCPO, Al, PS, StHy, and AA), the green areas represent moderately dense projections (En, VP, SI, VDB, HDB, LSI, TS, MS, PLd, VOLT, StA, AVPe, Ce, Me, BMA, BNST, EA, ACo, and AHi), and the blue areas represent sparsely dense projections (B, LSD, SHi, Ld, BMP, I, IPAC, PLCo, and PMCo; Figure 9).

## Discussion

**Figure 10:**
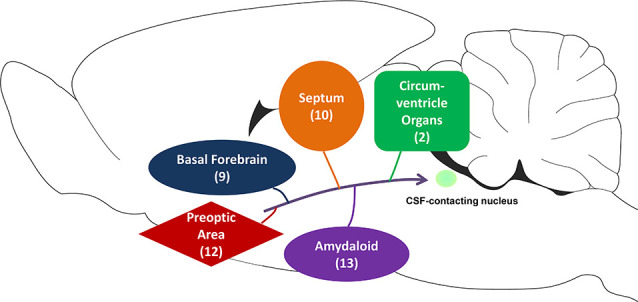
The schematic diagram of projections from the subcortex and limbic system to the CSF-contacting nucleus. Among them, basal forebrain contains nine sub-regions, septum contains 10 sub-regions, circumventricular organs contain two sub-regions, the preoptic area contains 12 sub-regions, and amygdaloid contains 13 sub-regions.

This present study systematically reveals that the CSF-contacting nucleus receives projections from the subcortex and limbic system (Figure 10). Many basic and clinical studies have focussed on these areas and reported some of their functions. Therefore, the biological functions of the CSF-contacting nucleus can be predicted according to their connection patterns in these regions.

### Functional Implications

#### Emotion

The CSF-contacting nucleus receives extensive projections from the septum and amygdaloid structures, which may participate in the modulation of emotions. The septum, a key component of the limbic system (Talishinsky and Rosen, 2012), is not a homogeneous structure (Risold and Swanson, 1997). It can be divided into different subregions. The lateral septal nuclei participate in depression-related behavior and are supposed to be a target for antidepressant drugs (Contreras et al., 1989, 2018). The MS has also been suggested to be a subcortical node in the modulation of anxiety (Zhang et al., 2017), as its ablation or pharmacological inhibition reduced the anxiety-like behavior of rats (Menard and Treit, 1996; Lamprea et al., 2010). The amygdaloid structures play important roles in emotional perception and expression (Shirasu et al., 2011). In anxiety disorders, the activities of the amygdaloid structures are significantly changed (Shin and Liberzon, 2010). The Ce in the amygdala has been ascribed an important role in the aversive states and behavioral dysregulation associated with stress (Gilpin et al., 2015). The neurons in the Me are activated by fear conditioning, and lesions in the Me can significantly disrupt the fear behavior (Tsuda et al., 2015). The BNST, which participates in the formation of the forebrain unit, is described as the “extended amygdala” (Alheid and Heimer, 1988), implying that it plays a pivotal role in the regulation of anxiety and mood, as well as in the pathophysiology of mood disorders (Fitzgerald et al., 2018).

#### Cognition

The CSF-contacting nucleus receives input from the basal forebrain and amygdaloid structures, which may participate in cognition. The cholinergic neurons of the basal forebrain are functionally related to attention and cognition, and their degeneration is implicated in Alzheimer’s and Parkinson’s diseases (Bohnen and Albin, 2011). The MS, VDB, HDB, and B, which are areas that are rich in cholinergic neurons in the basal forebrain, are strongly associated with learning and memory processing (Reznikov et al., 2009; Gratwicke et al., 2013; McHugh et al., 2015).

Studies of rodents and primates have shown that the amygdala is required for fear learning (Antoniadis et al., 2009; Salzman and Fusi, 2010). The Ce of the amygdala is the main output of this region (Sah et al., 2003), where its pharmacological intervention significantly changed the memory processes of rats (Hasanein and Sharifi, 2015). Apart from bidirectionally regulating various anxiety-like responses (Mazzone et al., 2018), the BNST has diverse contributions to aversive learning and memory (Goode and Maren, 2017), integrating the information from the amygdala, hippocampus, and prefrontal cortex (Weller and Smith, 1982; McDonald et al., 1999; Goode and Maren, 2017).

#### Homeostasis

Our previous study showed that the CSF-contacting nucleus participates in sodium sensing and appetite (Xing et al., 2015). The CSF-contacting nucleus receives input from the septum and preoptic area and circumventricular organs, which may participate in the regulation of homeostasis. The lateral septal nucleus (LS) is involved in the control of feeding behavior and energy homeostasis (Sweeney et al., 2017). Intra-LS infusions of gamma-aminobutyric acid (GABA) or acetylcholine increased feeding, whereas glucagon-like 1 peptide infusions decreased feeding (Scopinho et al., 2008; Mitra et al., 2014; Terrill et al., 2016). The MS is involved in fluid control and electrolyte balance, where its activation induces water intake, antidiuresis, natriuresis, and pressor responses (Melo et al., 2015).

The preoptic area sends dense and extensive projections to the CSF-contacting nucleus. This area contains intrinsically thermosensitive neurons which can sense the brain temperature (Boulant, 2000). The preoptic area is also a key component within the hierarchical organization of the neural circuits, which controls the thermoeffector activity (Morrison, 2016). Apart from thermal homeostasis, the preoptic area also participates in fluid balance. Use of the neurotoxin, ibotenic acid to destroy neuronal cell bodies in the MnPO in rats abolished their ability to drink in response to systemic hypertonic saline (Cunningham et al., 1992). The LPO is also regarded as a functional region that participates in drinking behavior (Saad et al., 1996).

The circumventricular organs SFO and VOLT also send numerous projections to the CSF-contacting nucleus. These two organs are regarded as the major osmosensory sites within the brain because they lack the normal blood-brain barrier (Augustine et al., 2018). VOLT has a causal role in the regulation of drinking behavior (Augustine et al., 2018). The optogenetic and chemogenetic activation of SFO neurons drives immediate and robust drinking behavior (Betley et al., 2015; Oka et al., 2015; Nation et al., 2016).

#### Visceral Activity

The CSF-contacting nucleus receives input from the MS, VDB, HDB, LPO, MPA, Me, Ce, and BNST, which may participate in visceral/autonomic activity. The MS, VDB, and HDB have been shown to participate in cardiovascular regulation (Calaresu et al., 1976; Nasimi and Hatam, 2005; Tavares et al., 2007), where chemical stimulation of these regions produced depressor and bradycardic responses (Gelsema and Calaresu, 1987; Kirouac and Ciriello, 1997). Microinjection of glutamate into the LPO decreased the heart rate, whereas local microinjection of GABAergic agonists evoked the opposite response (Osaka, 2012). For the MPA, microinjection of CoCl_2_ (a non-selective synapse blocker) caused tachycardia without altering the mean arterial pressure (Fassini et al., 2017). The Me also participates in cardiovascular responses, where its electrical stimulation has been reported to induce a mean arterial pressure and heart rate. Also, microinjection of noradrenaline into the Me caused significant cardiovascular changes (Fortaleza et al., 2011). The Ce and BNST are involved in visceral hypersensitivity in visceral nociception (Su et al., 2015; Ide et al., 2018).

#### Pain and Addiction

The CSF-contacting nucleus participates in pain modulation (Wang et al., 2014; Liu et al., 2017; Zhou et al., 2017), and it receives several pain-related regions in the subcortex and limbic system. The MS and diagonal band complex is crucial for information processing and chronic pain behavior (Jiang et al., 2018). These brain regions are involved in nociception modulation, especially that of affective, motivational, and cognitive behaviors (Ang et al., 2017). Pharmacological and electrophysiological experiments have revealed that the MPO and LPO participate in pain control and in periaqueductal grey-mediated endogenous analgesia (Silva et al., 2004). The Ce in the amygdala is termed the “nociceptive amygdala” (Neugebauer, 2015), as extensive research has shown that Ce neurons are sensitized in different pain models (Li and Sheets, 2018).

Several regions that participate in drug or alcohol addiction send extensive projections to the CSF-contacting nucleus. The nucleus accumbens is composed of two main regions, the shell, and the core. Specifically, the AcbSh is involved in contextual control over the extinction and reinstatement of drug-seeking for various drug classes (Gibson et al., 2019). The VP is a major target of the nucleus accumbens (Creed et al., 2016). VP neurons respond greatly to reward and reward-predictive cues (Tindell et al., 2005), and lesions of the VP reduce the hedonic impact and motivation for reward (Cromwell and Berridge, 1993). Activation of the Ce by optogenetic stimulation generates an addiction-like preference for reward (Tom et al., 2019). Moreover, the BNST is critical for the reinstatement of drug-seeking behavior and has shown changes in plasticity during abstinence from extended drug abuse (Harris and Winder, 2018).

## Conclusion

In summary, this study mapped the novel projections from the subcortex and limbic system to the CSF-contacting nucleus. According to the projection patterns, we hypothesize that the nucleus participates in the modulation of emotion, cognition, homeostasis, visceral activity, pain, and addiction. The results of this study provide a neuroanatomical basis for the explanation of neural or body fluids changes under these life activities and also provide the basis for intervening in the CSF-contacting nucleus in the treatment of the neurologic diseases. In the future, the advanced neural circuit intervention methods will be applied to further study the mechanism of the CSF-contacting nucleus in the above life activities.

## Data Availability Statement

All datasets presented in this study are included in the article.

## Ethics Statement

The animal study was reviewed and approved by Committee for Ethical Use of Laboratory Animals of Xuzhou Medical University.

## Author Contributions

S-YS and L-CZ designed the study and prepared the manuscript. S-YS, X-MZ, J-HD, L-LL, C-JS, JH, and J-LC injected the tracer and applied immunofluorescence experiments. S-YS and L-CZ made the 3D reconstruction of the projections.

## Conflict of Interest

The authors declare that the research was conducted in the absence of any commercial or financial relationships that could be construed as a potential conflict of interest.
